# On the Muscles Which Open the Eustachian Tube

**Published:** 1853-04

**Authors:** Joseph Toynbee


### ANATOMY AND PHYSIOLOGY.

*On the Muscles which open the Eustachian Tube.* By Joseph Toynbee, Esq., F. R. S.—The author commenced by alluding to the opinion generally held by anatomists, viz:—That the guttural orifice of the Eustachian tube is always open, and that the air in the tympanum is constantly continuous with that in the cavity of the fauces. An examination of the guttural orifice of the tube in man and other animals has led the author to conclude, that, except during muscular action, this orifice is always closed, and that the tympanum forms a cavity distinct and isolated from the outer air. The muscles which open the Eustachian tube in man are the tensor and levator palati, and it is by their action, during the process of deglutition, that the tubes are ordinarily opened. That the act of swallowing is the means whereby the Eustachian tubes are opened, is shown by some experiments, of which the following may be cited:—If the mouth and nose be closed during the act of swallowing the saliva, a sensation of fulness or distension arises from the air, which is slightly compressed in the fauces, passing into and distending the tympanic cavities. Upon removing the hand from the nose, it will be observed that this feeling of pressure in the ears does not disappear, but it remains until the act of deglutition is again performed, while the nose is not closed. In this experiment the Eustachian tubes were opened during each act of deglutition; during the first act, while they were open, air was forced into the cavity of the tympanum by the contraction of the muscles of the fauces and pharynx, and the guttural orifices of the tubes remained closed until the second act of swallowing, which opened the tubes, and allowed the air to escape. That the act of deglutition opens the Eustachian tubes was inferred also from the custom usually adopted of swallowing while the descent in a diving-bell is performed; by this act the condensed air is allowed to enter the tympanum, and the sensation of pain and pressure in the ears is removed or entirely avoided. The author gives an account of the Eustachian tube and its muscles in mammalia, birds, and reptiles. In some mammalia the muscles opening the tubes appertain as in man to the palate; in others, this function is performed by the superior constrictor muscles of the pharynx. In birds it is shown that there is a single membranous tube into which the two osseous tubes open; this membranous tube is situated between, and is intimately adherent to, the inner surface of each pterygoid muscle, and by these muscles the tube is opened. The conclusion to which the author arrives respecting the influence of the closed Eustachian tubes, is, that the function of hearing is best carried on while the tympanum is a closed cavity, and that the analogy usually cited as existing between the ordinary musical instrument the drum and the tympanum, to the effect that in each it is requisite for the air within to communicate freely with the outer air, is not correct. On the contrary, the author shows that no displacement of the air is requisite for the propagation of sonorous undulations, and that, were the Eustachian tubes constantly open, these undulations would extend into the cavity of the fauces, there to be absorbed by the thick and soft mucous membrane, instead of being confined to the tympanic cavity, the walls of which are so peculiarly well adapted to the production of resonance, in order that they shall be concentrated upon the labyrinth.

In corroboration of the above views, the author states, that in cases of deafness, dependent simply upon an aperture in the membrana tympani, whereby the sonorous undulations are permitted to escape into the external meatus, the power of hearing has been greatly improved by the use of an artificial membrana tympani, made of very thin vulcanized India-rubber or gutta percha, which is so applied as again to render the tympanum a closed cavity.

